# Supplementary material for: Nutrient density of Bangladeshi foods and its application in planning diet for pregnant women
Source: PLoS One. 2024 Jan 17;19(1):e0296831. doi: 10.1371/journal.pone.0296831 (PMC10793916; doi:10.1371/journal.pone.0296831)
Supplement: S2 Appendix — (DOCX) [file pone.0296831.s002.docx]

S2 Appendix: List of foods for formulating a nutrient-adequate diet for pregnant women.

| Food group | Food list |
| --- | --- |
| Cereals, starchy roots, and tubers | Barley, White bread, Proso millet, Pear millet, Rice, Brown wheat flour, Whole wheat, Elephant foot, Giant taro, Potato, Orange flesh sweet potato. |
| Pulses and legumes | Bengal gram (dehulled), Bengal gram (whole dried), Black gram (dehulled), Cowpea, seed, Grass pea, Green gram (split dried), Green gram (whole), Lentil, Pea, Soybean. |
| Non-leafy vegetables | Amaranth, stem, Bean, Carrot, Cucumber, Ash gourd, Pointed gourd, Okra, Pumpkin, Radish, Onion stalk |
| Leafy vegetables | Red amaranth leaves, Spiney Amaranth leaves, Green amaranth, leaves, Beet greens leaves, Bengal dayflower leaves, Fenugreek leaves, Indian spinach, Jute leaves, Mustard leaves, Bathua leaves |
| Fruits | Fig, Hog plum, Jambolan, Fazli mango, Melon futi, Monkey-jack, Muskmelon, Bangee, Orange juice, Orange, Sweet orange. |
| Fish | Barb, Bronze featherback, Day's mystus, Ganges River sprat, Indian river shad, Minnow finescale razorbelly, Minnow, largescale razorbelly, Mola carplet, Rohu, Spotted snakehead |
| Meat, poultry, and egg | Beef liver, Beef meat, Beef mince, Chicken liver, Duck meat, Mutton liver, Duck liver, Other Birds meat (Turkey, Quail), Chicken eggs, Chicken egg yolk, Duck eggs. |
| Milk | Buttermilk, Cottage cheese, Sweetened curd, Buffalo milk, Powdered skimmed milk, Powdered whole milk, Skimmed cow milk, Whole fat (pasteurized, UHT) cow milk, Condensed milk, Goat milk. |
| Additional food | Soya oil |
