# Supplementary material for: Nutrient density of Bangladeshi foods and its application in planning diet for pregnant women
Source: PLoS One. 2024 Jan 17;19(1):e0296831. doi: 10.1371/journal.pone.0296831 (PMC10793916; doi:10.1371/journal.pone.0296831)
Supplement: Attachment — Submitted filename: Reviewing notes PONE-D-23-13289.docx [file pone.0296831.s003.docx]

PLOS ONE

Manuscript Number: PONE-D-23-13289

Nutrient Density of Bangladeshi foods and its application in diet planning for pregnant women

Since the paper involves a large number of abbreviations for simplicity, it is recommended that a list of such abbreviations be included.

**Nutrient Adequacy Score (NAS)**

**Naturally nutrient-rich score 7 (NNR7)**

**Recommended dietary allowances (RDA)**

**being small for gestational age (SGA)**

**Food Composition Table of Bangladesh (FCTB)**

**Food-based dietary guidelines of Bangladesh (FBDG)**

**Linear programming (LP)**

**Estimated energy requirement (EER)**

**Physical activity level (PAL)**

**Tolerable Upper Limit (TUL)**

**Acceptable Macronutrient Distribution Range (AMDR)**

It is necessary that each equation be assigned a number.

Folic acid deficiency is typically regarded to be more common than calcium deficiency among pregnant women Inadequate folic acid intake during pregnancy is connected with a number of potential health problems. What are these risks? Why the authors were folic acid excluded from the study, even though the need for folic acid is important at all stages of pregnancy? As folic acid is no less important than calcium, which was mentioned in this study.

In line 26 NNR7,98, 108, 238, 246, 308, 318 NNR7, these terms new naturally nutrient-rich score, Naturally Nutrient Rich 7, new Nutrient-Rich Foods (NRF), and nutrient density scoring matrix are not new and you should specify with new metric for pregnant women in Bangladesh.

Actually, these terms had been used in various research studies and publications to assess the nutritional quality of different foods and dietary patterns. The United States Department of Agriculture (USDA) created a nutrient profiling technique known as the Nutrient-Rich Foods Index in order to evaluate the overall nutritional content of different foods. It assigns a ranking to each food item based on its nutrient density, considering both the good and negative aspects of the foods' nutrient composition when doing so.

In line 27, the phrase “third trimester pregnant women” should be added to the “specifically for pregnant women…”.

In line 62, remove the short letters of the being small for gestational age (SGA) as it didn’t repeat it within entire manuscript.

In line 100, the authors only targeted the later stages of pregnancy; they did not target the entire gestation period. Therefore, only referring to pregnant women in their third trimester would be the most accurate course of action to pursue.

In line 115, delete “and” of the and egg.

In line 117-120, the sentence is needs reference (such as FAO or FANTA,...etc).

In line 134 table 1, the references should be mention the recommended dietary allowance of pregnant women and which allowances were followed (icmr-NIN and/or others).

In line 146, are the authors followed a specific food group classification. The classification of food groups can vary depending on the system or organization you're should referring to! However, one common classification system is based on the United States Department of Agriculture (USDA) food groups, which are used for dietary guidelines and nutritional recommendations.

In line 163, the word light should be change with sedentary.

In line 172, the phrase ‘adverse effects on excess consumption’ is belong to the pregnant women take any supplements or medication or from the natural food? What grad of the adverse effect from natural food? I would suggest to remove these phrases or justified.

In line 223, in the table 4 authors should mention to the Indian icmr-NIN as they considered as a RDA reference

In line 224 table 4, food groups. Thiamine is a vitamin that can be degraded when exposed to high temperatures since it is heat-sensitive. However, the amount of thiamine that actually breaks down during baking might be somewhat variable. Such as... * Note: Thiamine is sensitive to heat; therefore, grains and baked goods are not considered the primary sources of thiamine.
